# Vascular Endothelial Cell-Specific Connective Tissue Growth Factor (CTGF) Is Necessary for Development of Chronic Hypoxia-Induced Pulmonary Hypertension

**DOI:** 10.3389/fphys.2018.00138

**Published:** 2018-02-27

**Authors:** Liya Pi, Chunhua Fu, Yuanquing Lu, Junmei Zhou, Marda Jorgensen, Vinayak Shenoy, Kenneth E. Lipson, Edward W. Scott, Andrew J. Bryant

**Affiliations:** ^1^Department of Pediatrics, University of Florida, Gainesville, FL, United States; ^2^Division of Pulmonary, Critical Care, and Sleep Medicine, Department of Medicine, College of Medicine, University of Florida, Gainesville, FL, United States; ^3^Department of Pharmaceutical and Biomedical Sciences, California Health Sciences University, Clovis, CA, United States; ^4^FibroGen, Inc., San Francisco, CA, United States; ^5^Department of Molecular Genetics and Microbiology, University of Florida, Gainesville, FL, United States

**Keywords:** pulmonary hypertension, hypoxia, connective tissue growth factor (CTGF), CD11b/integrin α_M_ (ITGAM), cell division control protein 42 homolog (Cdc42)

## Abstract

Chronic hypoxia frequently complicates the care of patients with interstitial lung disease, contributing to the development of pulmonary hypertension (PH), and premature death. Connective tissue growth factor (CTGF), a matricellular protein of the Cyr61/CTGF/Nov (CCN) family, is known to exacerbate vascular remodeling within the lung. We have previously demonstrated that vascular endothelial-cell specific down-regulation of CTGF is associated with protection against the development of PH associated with hypoxia, though the mechanism for this effect is unknown. In this study, we generated a transgenic mouse line in which the *Ctgf* gene was floxed and deleted in vascular endothelial cells that expressed Cre recombinase under the control of VE-Cadherin promoter (eCTGF KO mice). Lack of vascular endothelial-derived CTGF protected against the development of PH secondary to chronic hypoxia, as well as in another model of bleomycin-induced pulmonary hypertension. Importantly, attenuation of PH was associated with a decrease in infiltrating inflammatory cells expressing CD11b or integrin α_M_ (ITGAM), a known adhesion receptor for CTGF, in the lungs of hypoxia-exposed eCTGF KO mice. Moreover, these pathological changes were associated with activation of—Rho GTPase family member—cell division control protein 42 homolog (Cdc42) signaling, known to be associated with alteration in endothelial barrier function. These data indicate that endothelial-specific deletion of CTGF results in protection against development of chronic-hypoxia induced PH. This protection is conferred by both a decrease in inflammatory cell recruitment to the lung, and a reduction in lung Cdc42 activity. Based on our studies, CTGF inhibitor treatment should be investigated in patients with PH associated with chronic hypoxia secondary to chronic lung disease.

## Introduction

Hypoxia is traditionally held to be a necessary factor in the pathogenesis of pulmonary hypertension (PH) secondary to chronic lung disease, classified as World Health Organization (WHO) Group III PH (Klinger, 2016). This contribution is clinically important, as Group III PH patients have at least a four-fold increased risk of death compared to disease controls without pulmonary vascular disease (Lettieri et al., 2006), and no proven treatments exist. Thus, studies of novel molecular pathways contributing to disease are needed in order to identify therapeutic targets.

To this end, the tissue response to low oxygen mediated by hypoxia-inducible factor (HIF)—a tightly conserved and regulated transcription factor across species—is known to be integral in the development of PH (Yu et al., 1999; Brusselmans et al., 2003). Building on these data, our group has recently uncovered a necessary role for tissue-specific—vascular endothelium derived—HIF in several models of PH (Bryant et al., 2015, 2016). Specifically, we found that deletion of endothelial HIF leads to improved vascular barrier integrity in response to injury, and is associated with protection against pulmonary vessel “leakiness” leading to PH. These findings echo previously reported results that described normalization of pulmonary pressures, in a genetic model of PH, associated with a decrease in endothelial layer permeability (Burton et al., 2011a). Of note, protection against these lung vasculopathic changes was in part due to reduced extravasation of circulating myeloid cell mediators into the pulmonary vascular bed (Burton et al., 2011b), although an exact mechanism is a topic of ongoing debate.

Therefore, a primary objective was to find a candidate targetable hypoxia-response element gene that is both (1) a protein involved in cell-cell adhesion and remodeling, and (2) a chemoattractant for circulating myeloid cells. Significantly, we discovered that connective tissue growth factor (CTGF)—a matricellular protein of the CCN family important for cell-cell and cell-matrix crosstalk (Lipson et al., 2012)—is upregulated in an endothelial cell autonomous fashion during hypoxia exposure (Bryant et al., 2016). Notably, CTGF is an identified coordinator of vascular repair (Pi et al., 2011), and a known ligand for the integrin α_M_ (ITGAM)—also known as CD11b—adhesion receptor expressed by peripheral blood leukocytes (Schober et al., 2002). Based on these findings, we asked the question: what is the mechanistic role of CTGF in pulmonary vascular remodeling? Our central hypothesis, based upon this query, is that endothelial CTGF is necessary for recruitment of CD11b expressing leukocytes to the lung, and development of PH. In this study, we demonstrate in two models of PH, that endothelial CTGF is necessary for development of disease, through accumulation of CD11b^+^ cells and downstream factors promoting aberrant vascular repair.

## Materials and methods

### Reagents, antibodies, and primers

FG-3019 (10 mg/kg) and control IgG (10 mg/kg)—both administered intraperitoneal every week at initiation of hypoxia exposure (Wang et al., 2011)—were the kind gift of FibroGen Inc. (San Francisco, CA). Bleomycin Sulfate, ML141, and dichloromethylenediphosphonic acid disodium salt (clodronate) was purchased through MilliporeSigma. Lipids and cholesterol for liposome production were bought from Avanti Polar Lipids. EBM-2 culture media (Lonza), with EGM-2 bulletkit, was utilized for all *in vitro* experiments. Primer sequences are as follows: CTGF forward primer GGGAGAACTGTGTACGGAGC; CTGF reverse primer AGTGCACACTCCGATCTTGC; CD11b forward primer ATGGACGCTGATGGCAATACC; CD11b reverse primer TCCCCATTCACGTCTCCCA; 18S forward primer ACCTGGTTGATCCTGCCAGTAG; and, 18S reverse primer TTAATGAGCCATTCGCAGTTTC. Antibodies used in this study were: anti-GFP (Aves), MECA-32 (BD Biosciences), CTGF (BD Biosciences), CD11b (Abcam), F4/80 (BD Biosciences), CD31 (Santa Cruz Biotechnology), and GAPDH (Abcam). Antibodies for flow cytometry used in this study were: CD45 (FITC; BioLegend), CD11b (APC-Cy7; BioLegend), and IgG2 (isotype control; BioLegend). Active Cdc42 detection kit was purchased through Cell Signaling Technology. Secondary fluorescent antibodies were from Jackson Immunoresearch. Refer to Supplementary Table 1 for full details of antibody catalog number and dilutions.

### Animals

All wild-type (Jackson Laboratory) and transgenic mice generated for this study were on the C57BL/6J background, were greater than 8 weeks of age at the study onset, included both males and females, and ranged in weight from 20 to 30 g. Transgenic mice expressing Cre-recombinase under control of the mouse VE-Cadherin promoter (VECad.Cre) (Alva et al., 2006) were crossed with mice in which exon 4 was flanked by two loxP sites (*Ctgf* ^fl^) to create Cre mediated deletion the *Ctgf* gene within vascular endothelium (*Ctgf* ^fl/fl^, VECad-Cre). Breeding pairs were set up such that *floxed* Ctgf allele was maintained in homozygous state while VECad-Cre was in the heterozygous state, yielding Cre-positive mice with endothelial deletion of CTGF while *Ctgf* ^fl/fl^ mice served as littermate controls. To control for potential Cre-positive effects, in some experiments, VECad.Cre mice were used as part of the control cohort. Experiments to identify CTGF-expressing cells used CTGF-GFP mice, in which transgenic expression of green fluorescent protein (GFP) is driven by the *Ctgf* promoter (Pi et al., 2011). Mice were housed in the central animal care facility at University of Florida College of Medicine (Gainesville, FL) and were given food and water *ad libitum*. The experimental protocol was reviewed and approved by the institutional animal care and utilization committee at University of Florida.

### Chronic hypoxia and bleomycin models

Mice exposed to chronic hypoxia were placed in a normobaric chamber (Coy Laboratory Products) with continuous monitoring of oxygen and carbon dioxide concentration, ensuring that ventilation was maintained such that carbon dioxide levels remain < 0.1%. Mice were housed in the same room under normoxia (room air, FiO_2_ 21%) or hypoxia (FiO_2_ 10%) for a period of 4 weeks. At the completion of the chronic hypoxia protocol, mice underwent harvest for histology and hemodynamic measurement (Bryant et al., 2016). In indicated experiments, a separate group of mice underwent intraperitoneal injection with 0.018 U/g bleomycin (Thermo Fisher Scientific) or vehicle twice weekly for 28 days (Baran et al., 2007; Bryant et al., 2015). A group of vehicle- or bleomycin-treated mice were concomitantly dosed with 100 μL intraperitoneal liposomal clodronate (CL_2_MDP) 1 week prior to either bleomycin or chronic hypoxia exposure, and every 3 days thereafter, in order to induce lung macrophage apoptosis. Clodronate liposomes, and control PBS liposomes, were generated as previously described (Murray et al., 2011; Zaynagetdinov et al., 2013). One week after the last injection, mice were then harvested for histology and hemodynamic measurements, similar to hypoxia-treated groups. ML141 (5 mg per kg body weight), a Cdc42 inhibitor, was dissolved in dimethyl sulfoxide (DMSO), and was intraperitoneally injected daily throughout bleomycin administration protocol (Chen et al., 2015), in described experiments.

### PH assessment

Invasive hemodynamic measurement was conducted, as described previously (West et al., 2008). In brief, upon induction of a surgical plane of anesthesia with avertin (500 mg/kg, intraperitoneal) the right internal jugular vein was exposed. The distal portion of the vein was completely occluded, while the proximal end of the jugular was loosely tied. A small incision was made in the exposed vein, and a Millar 1.4 French pressure-volume microtip catheter transducer (SPR-839; Millar Instruments, Houston, TX) connected to a PowerLab/8s (ADInstruments) was inserted through the incision and threaded down into the right ventricle. Data were collected using Chart 5 (ADInstruments). The heart was then excised with removal of the atria, and the RV and left ventricle (LV) plus septum were isolated for measurement of the RV:LV+S as previously described (Hemnes et al., 2014).

### Isolation of pulmonary microvascular endothelial cells (PMVECs)

Primary isolates of PMVECs were obtained from transgenic mice and littermate controls, as previously described (West et al., 2014). In brief, cells were prepared from uninjured mice using collagenase type 2 and red blood cell lysis buffer. Endothelium was then isolated by fluorescence-activated cells sorting (FACS) based on CD31-PECAM-1 expression. To induce endothelial differentiation, sorted cells were plate on gelatin-coated plastic and were cultured in endothelial growth medium (Lonza, Walkersville, MD), until cells were confluent. Cells were then incubated with Alexa 488-labeled AcDiLDL (Life Technologies, Grand Island, NY) as previously described (Irwin et al., 2007), and positively stained cells were enriched by flow cytometry. Cells were then expanded and phenotyped as described.

### Flow cytometry

Flow cytometry analyses were performed on a BD LSR II or on FACSCalibur upgraded at three lasers and 8 colors (Cytek). Cell populations were identified using sequential gating strategy characterized within body of manuscript (excluding debris and doublets). Fluorescence minus one (FMO) and isotype controls were used when necessary. The expression of markers is presented as median fluorescence intensity (MFI). Data were analyzed using FlowJo software (Tree Star).

### Immunohistochemical, RNA, and protein analyses

Upon harvest, the left lobe of the lung was inflated and placed in either 10% formalin or 4% PFA followed by sucrose for histological processing as previously described (Degryse et al., 2011; Tanjore et al., 2013), and the right lobes were snap frozen in liquid nitrogen for RNA and protein processing. Sections were prepared and processed for immunohistochemistry and immuno-fluorescence staining as previously described (Tanjore et al., 2009). Semiquantitative lung fibrosis scoring (10) and hydroxyproline microplate assay were performed, as previously described (11). For morphometric measurement, CTGF expression was analyzed quantitatively based on green fluorescence intensity from CTGF-GFP transgenic animals and CD11b expression was based on red fluorescence intensity from eCTGF KO lungs. At least 10 images (20x magnification)/lung sections were analyzed using ImageJ software (http://rsb.info.nih.gov/ij/). Relative expression was calculated as a percentage of total pixel intensity in each image. Western blots were performed on tissue and cell lysates as previously described (Tanjore et al., 2011). Total RNA was isolated from frozen whole-lung tissue and human cell lysate using a RNEasy kit (Qiagen) per manufacturer recommendations, DNase treated and prepared for quantitative RT-PCR according to previous report (Pi et al., 2015). Specific transcript levels for target genes were then determined by normalization to 18s. Values are presented as mean normalized transcript level using the comparative *C*_*t*_ method (2^−ΔΔ*Ct*^).

### Statistical analyses

Statistical analysis was performed using GraphPad Prism 6.0 (GraphPad Software). Data are expressed as means ± SEM. As appropriate, groups were compared by ANOVA; follow-up comparisons between groups were conducted using Student's *t*-test. A *P* ≤ 0.05 was considered to be significant.

## Results

### Blockade of CTGF in hypoxia model rescues PH phenotype

**Figure 1:**
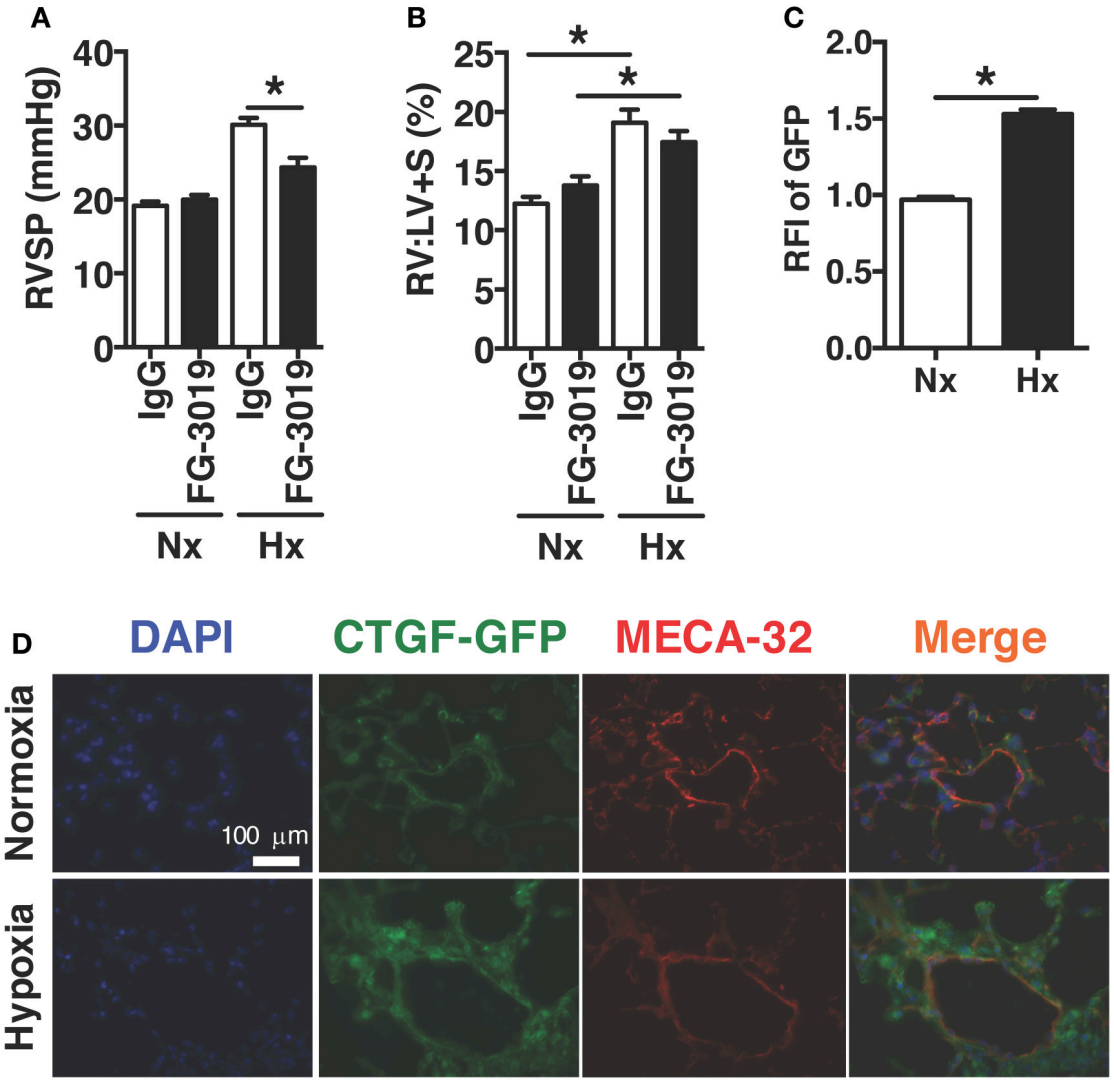
Pharmacological inhibition of CTGF protects mice from hypoxia-induced PH. **(A)** Right ventricular systolic pressure (RVSP) and **(B)** Right ventricle to left ventricle plus septal mass ratio (RV:LV+S) in control IgG- or FG-3019-treated mice, exposed to normoxia (FiO_2_ 21%) or chronic hypoxia (FiO_2_ 10%; day 28, *n* = 8 mice/group). Dose for both FG-3019 and IgG was 10 mg/kg, injected intraperitoneally, once weekly. **(C)** Quantification of GFP relative fluorescent intensity (RFI) and **(D)** representative immunofluorescent images (40x magnification; scale bar 100 μm) from CTGF-GFP mice exposed to normoxia or hypoxia, also co-stained for vascular endothelial cell marker, MECA-32. ^*^P < 0.05; data are presented as mean ± SEM.

In order to investigate the therapeutic targeting of CTGF in chronic hypoxia induced PH, we determined whether the pulmonary vascular response could be suppressed by systemic administration of a CTGF inhibitory monoclonal antibody, FG-3019. FG-3019 was therefore administered by intraperitoneal injection three times weekly starting the day prior to the onset of hypoxia exposure, in a preventive regimen. The extent of PH, as measured by right-ventricular systolic pressure (RVSP; mmHg), was significantly reduced in those mice treated with FG-3019, as compared to control IgG treated mice (Figure 1A). Additionally, the extent of protection against PH development by FG-3019 was also quantified by assessing the ratio of right ventricle to left ventricle plus septal mass (RV:LV+S; %), a surrogate marker of right ventricular remodeling in response to PH. Though there was a trend toward a decrease in RV remodeling, there was no statistically significant difference between hypoxia-exposed groups. Collectively, these findings suggested that an intrinsic lung vascular change accounted for differences in pressures (Figure 1B). In order to evaluate this vascular specific effect further we performed co-immunofluorescent staining for MECA32 (highlighting the vascular endothelium) in CTGF-GFP reporter mice—in which GFP expression is driven by the *Ctgf* promoter—exposed to normoxia vs. hypoxia. We found that the vascular endothelium significantly increased expression of CTGF in lung sections from hypoxia vs. normoxia animals (Figures 1C,D). Taken together, these data indicate that CTGF importantly contributes to the development of PH, and that FG-3019 can suppress PH—in the described murine models—through CTGF-inhibition.

### CTGF expression is enhanced in hypoxia model in lung CD11b^+^ cells

**Figure 2:**
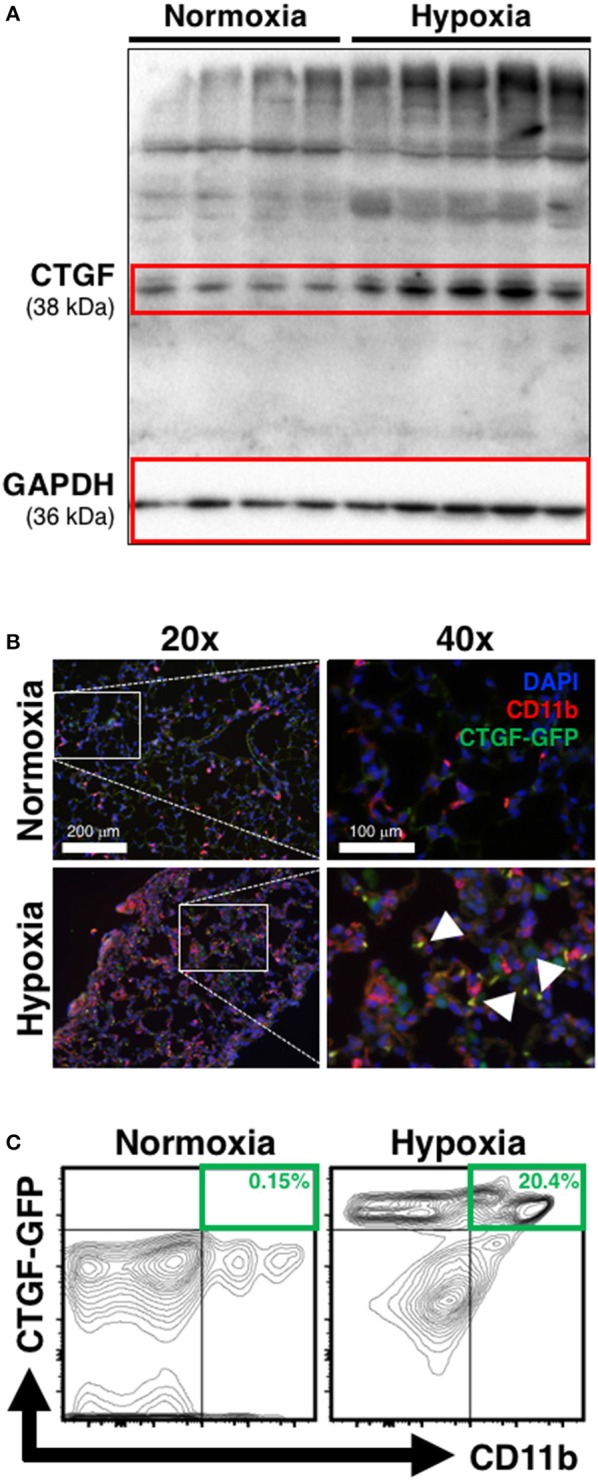
Chronic hypoxia-induced PH is associated with CTGF- and CD11b-expression within the lung. **(A)** Western blot on whole lung of mice exposed to normoxia vs. chronic hypoxia (*n* = 4 and 5 mice/group, respectively). **(B)** Representative CD11b (white arrowheads) immunofluorescent staining of lung section from CTGF-GFP reporter mice exposed to either normoxia or hypoxia (20x and 40x magnification; scale bars 100 and 200 μm, respectively). **(C)** Representative flow cytometry contour plots detailing GFP and CD11b co-expression of whole lung CD45^+^ cells, after normoxia or hypoxia treatment for 28 days, with percentages listed (*n* = 8–10 mice/group).

The induction of CTGF under hypoxic conditions has been recognized as a key contributor to conditions of aberrant tissue remodeling (Higgins et al., 2004). Likewise, CTGF-expression is known to contribute to perivascular myeloid cell accumulation in the pathology of atherosclerotic arterial changes in cardiac disease (Schober et al., 2002). Therefore, we hypothesized that CTGF expressed during chronic hypoxia exposure critically influences accumulation of CD11b^+^ cells in the lung. To investigate the contribution of CTGF to CD11b^+^ cell accrual during the development of PH, we first confirmed global lung increase in CTGF expression in response to chronic hypoxia (Figure 2A). In CTGF-GFP mice, lung-sections co-stained for CD11b exhibited co-localization with CTGF that was qualitatively increased in hypoxia compared to normoxia exposed mice (Figure 2B). This is consistent with either increased binding of CTGF to CD11b^+^ cells, or to increased expression of CTGF in the CD11b-expressing cells, though the latter is less likely since CD11b-expressing leukocytes are known to produce little CTGF intrinsically (Cicha et al., 2005). Flow cytometric assessment confirmed that the percentage of dual positive CTGF and CD11b cells in the lungs was increased in hypoxia (20.4% of CD45^+^ cells) vs. normoxic (0.15% of CD45^+^ cells; Figure 2C) controls. These data suggest that CTGF contributes to the expansion of the CD11b^+^ myeloid cell pool within the lung, possibly through direct interaction with CD11b upon exposure to hypoxia, since CTGF is a ligand for this adhesion receptor that is encoded by the *ITGAM* gene on leukocytes (Schober et al., 2002).

### Vascular endothelial CTGF is necessary for development of PH secondary to hypoxia

**Figure 3:**
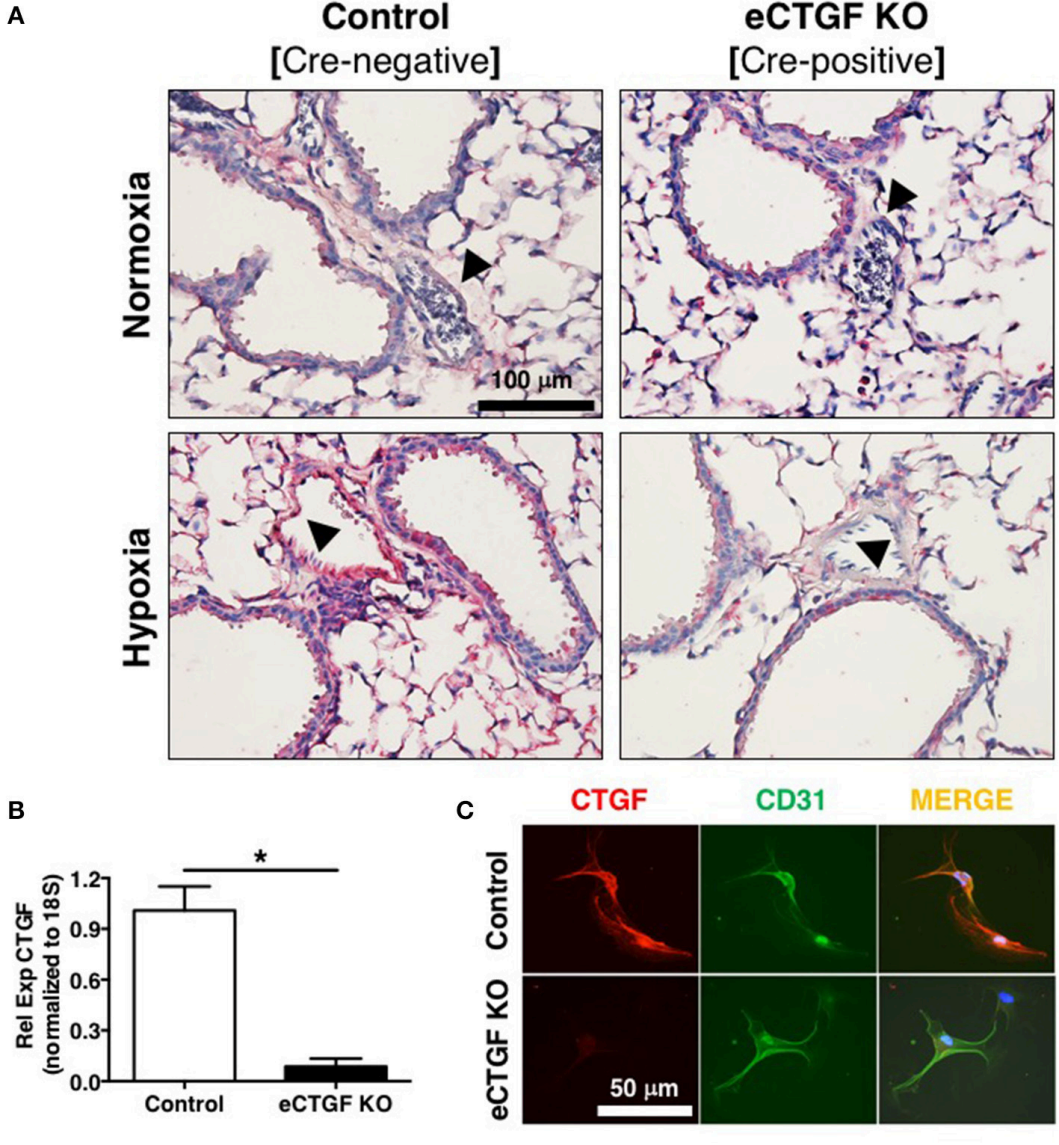
Mice designated eCTGF KO (VECadherin.Cre-CTGF^fl/fl^) have no CTGF expression in pulmonary vascular endothelial cells. **(A)** Representative immunohistochemical stain for CTGF (red) in whole lung sections from Control and eCTGF KO mice, upon exposure to normoxia or hypoxia. **(B)** qPCR for CTGF mRNA (*n* = 4 mice/group) and **(C)** immunofluorescent double staining for CD31 and CTGF proteins, in pulmonary microvascular endothelial cells isolated from wild type (WT) and eCTGF KO mice (200x magnification, scale bar 50 μm). ^*^P < 0.05; data are presented as mean ± SEM.

**Figure 4:**
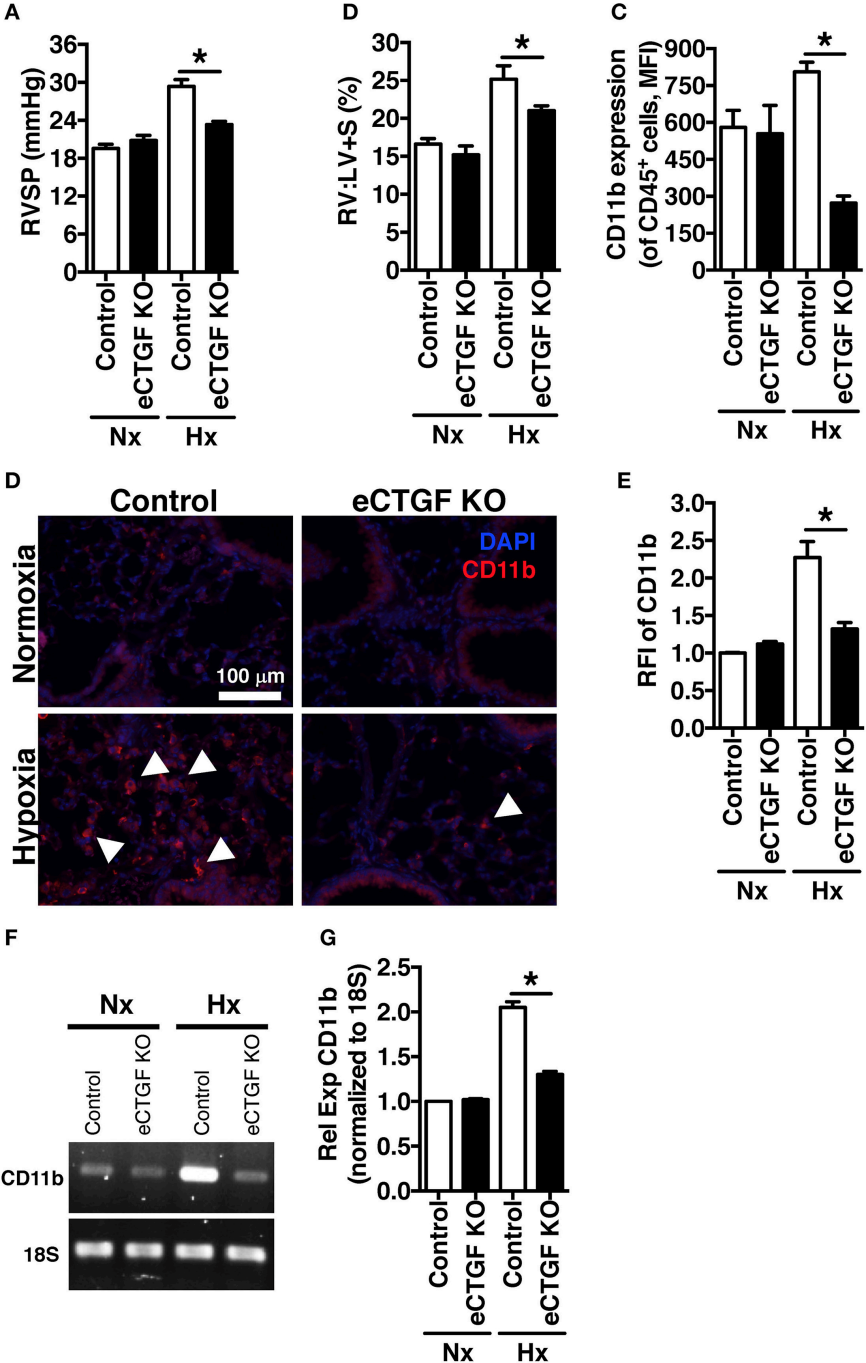
The development of chronic-hypoxia induced PH is dependent upon pulmonary vascular endothelial cell expression of CTGF, and CD11b^+^ cell accumulation within the lung. **(A)** RVSP and **(B)** RV:LV+S in Control and eCTGF KO mice, exposed to normoxia or chronic hypoxia (*n* = 10 mice/group). **(C)** CD11b-expression (MFI) as determined by flow cytometric analysis of CD45^+^ cells from lungs of mice treated with either normoxia or hypoxia (*n* = 8 mice/group); **(D)** Representative immunofluorescent images of lung sections from described groups, staining for CD11b (40x magnification; scale bar 100 μm), and **(E)** quantification of CD11b relative fluorescent intensity (RFI). **(F,G)** PCR for CD11b-expression in whole lung of mice in indicated groups. ^*^P < 0.05; data are presented as mean ± SEM.

Based on our lab's previous work demonstrating alteration in vascular endothelial cell CTGF as associated with PH pathophysiology (Bryant et al., 2015, 2016), we next sought to evaluate the impact that endothelial cell CTGF deletion would have on disease progression. In order to test our central hypothesis, we developed a genetic mouse model in which *Ctgf* ^fl/fl^ alleles were deleted by the Cre recombinase under the control of the VECadherin-promoter, creating endothelial specific knockouts (eCTGF KO). Immunohistochemical staining for CTGF expression corroborated that in eCTGF KO mice, CTGF was not expressed in the thin vascular endothelial cells lining blood vessels within the lung after hypoxia exposure (Figure 3A). In addition, we examined isolated pulmonary microvascular endothelial cells (PMVECs) from our genetic model, for RNA (Figure 3B) expression level confirmation that CTGF was not present in cells compared to controls. We also found little to no CTGF staining in isolated PMVECs by immunofluorescence, co-stained with CD31 (Figure 3C). Next, we exposed our eCTGF KO mice to chronic hypoxia, and found that they were protected against development of PH (Figure 4A), with an associated decrease in RV remodeling (Figure 4B). The decrease in RVSP was associated with a decrease in leukocyte CD11b expression within the lung, as assessed by flow cytometry (Figure 4C). This was reinforced qualitatively, and semi-quantitatively, by examining CD11b immunofluorescent staining of lung sections, demonstrating fewer CD11b^+^ cells in the lungs of eCTGF KO mice after hypoxia (Figures 4D,E). RNA level expression of CD11b in whole lung was likewise decreased in eCTGF KO mice after hypoxic exposure, compared to controls (Figures 4F,G). Taken together, these data demonstrate the critical role of endothelial CTGF to the pulmonary vascular response to chronic hypoxia, associated with increased CD11b-expression.

### Endothelial CTGF expression is necessary in a bleomycin model of PH

**Figure 5:**
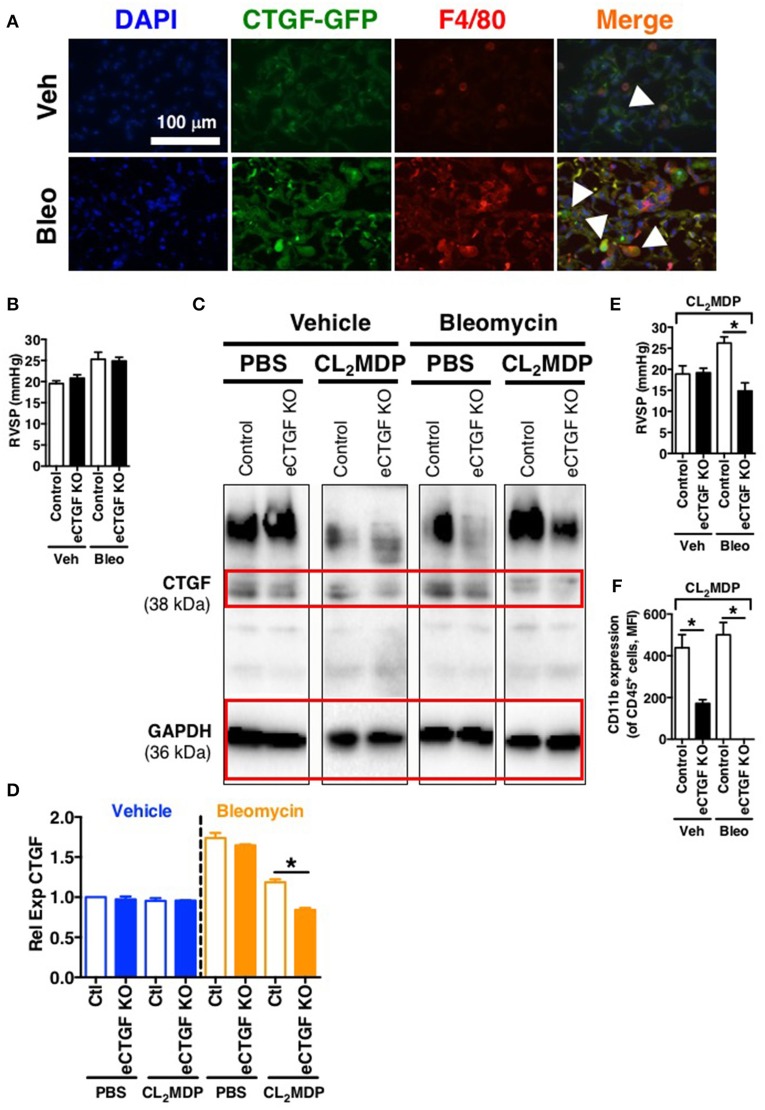
Vascular endothelial cell CTGF production induced in a bleomycin model of PH is necessary for development of elevated pulmonary pressures. **(A)** Representative immunofluorescent images (40x magnification; scale bar 100 μm) from CTGF-GFP mice exposed to normoxia or hypoxia, co-stained for macrophage marker, F4/80. **(B)** RVSP measurement in Control and eCTGF KO mice, exposed to vehicle (PBS) or intraperitoneal bleomycin (day 33; *n* = 8 mice/group). **(C)** Western blot and **(D)** densitometry analysis on whole lung of Control and eCTGF KO mice detailing CTGF expression upon exposure to bleomycin and either PBS- or clodronate (CL_2_MDP) liposomes. **(E)** RVSP in Control and eCTGF KO mice, exposed to CL_2_MDP liposomes and either vehicle (PBS) or intraperitoneal bleomycin (day 33; *n* = 8–10 mice/group). **(F)** CD11b-expression, by flow cytometry, of CD45+ cells from lungs of treated mice (*n* = 8 mice/group). ^*^P < 0.05; data are presented as mean ± SEM.

In order to establish rationale for the following series of experiments, we must consider possible cell sources of CTGF. CTGF is known to be increased in whole lung of patients with chronic lung disease (Noguchi et al., 2017). In pulmonary fibrosis, CTGF is expressed in interstitial fibroblasts and type 2 alveolar epithelial cells (Pan et al., 2001). In addition to endothelial cells, other vascular cells can express CTGF, including pericytes (Suzuma et al., 2000) and vascular smooth muscle cells (Lee et al., 2005). Likewise, in hepatic fibrosis models, production of CTGF has been shown to be a necessary factor in progression of scarring of chronic fibrosis, and co-localization of CTGF was observed with liver macrophages/Kuppfer cells in a biliary fibrosis model (Pi et al., 2015). Importantly, modest expression of CTGF in myeloid cells has also been observed in other models. For example, *in vitro* CTGF expression in monocyte-derived macrophages can be induced (Ikezumi et al., 2015), and modest CTGF expression was observed in THP-1 cells polarized to M1, M2a or M2c macrophage phenotypes, with much more robust expression if the polarized macrophages were co-cultured with a mesenchymal cell population (Finlin et al., 2013). Similarly, we found in the intraperitoneal bleomycin model of pulmonary fibrosis an increase in CTGF expression by F4/80^+^ macrophages within the lung (Figure 5A). The fact that these non-endothelial cells express CTGF may explain, in part, our findings that after chronic intraperitoneal bleomycin exposure alone (twice weekly for 33 days), eCTGF KO mice were not protected against development of PH (Figure 5B), or pulmonary fibrosis (Supplementary Figure 1). Intriguingly, our group has recently described a novel model for PH, administering bleomycin in combination with clodronate (CL_2_MDP, in figures) liposomes, resulting in depletion of macrophages and robust PH, with nearly no evidence of pulmonary fibrosis (Bryant et al., 2017). In light of these findings, we hypothesized that depletion of lung phagocytes with clodronate liposome administration would lead to decreased whole lung CTGF-expression, with a greater dependence on CTGF from the vascular/endothelial compartment in maintaining the wound regulatory balance. Given this rationale, we found an appreciable decrease in CTGF in lungs of mice treated with clodronate, compared to PBS liposome controls (Figures 5C,D). Using this model, we were next able to confirm the main findings of the chronic hypoxia experiments, validating that eCTGF KO mice were protected against development of PH (Figure 5E). The normalization of pulmonary pressures was also shown to correlate with a decrease in CD45^+^ cell CD11b expression (Figure 5F). These results suggest that endothelial CTGF is integral in recruited myeloid-cell associated pulmonary vascular remodeling in multiple models of PH.

### Endothelial CTGF expression activates Cdc42 in lungs of mice exposed to hypoxia and bleomycin, while Cdc42 inhibition protects against development of PH

**Figure 6:**
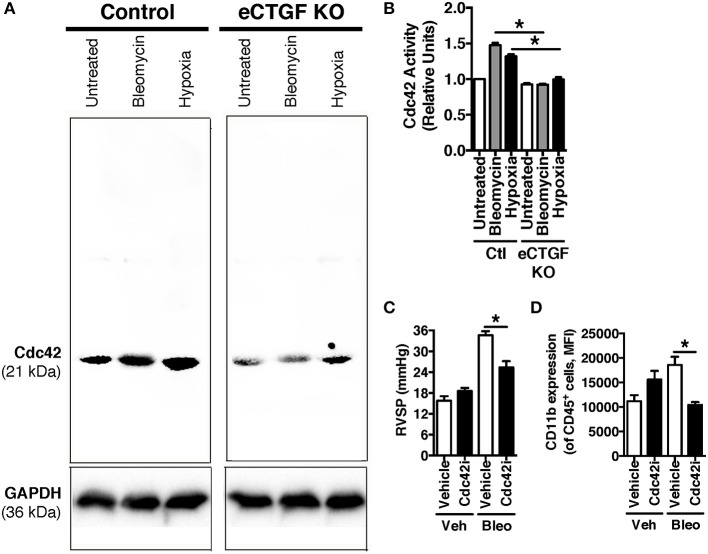
Inhibition of Cdc42 suppressed PH in response to chronic hypoxia. **(A,B)** Cdc42 activation under normoxic/vehicle-, bleomycin-treated, and hypoxic conditions in lungs of Control and eCTGF KO mice. **(C)** RVSP in vehicle or Cdc42 inhibitor-treated (ML141; indicated as “Cdc42i”) mice exposed to either vehicle or intraperitoneal bleomycin (*n* = 8 mice/group). **(D)** CD11b-expression, by flow cytometry, of CD45+ cells from lungs of treated mice (*n* = 8 mice/group). ^*^P < 0.05; data are presented as mean ± SEM.

**Figure 7:**
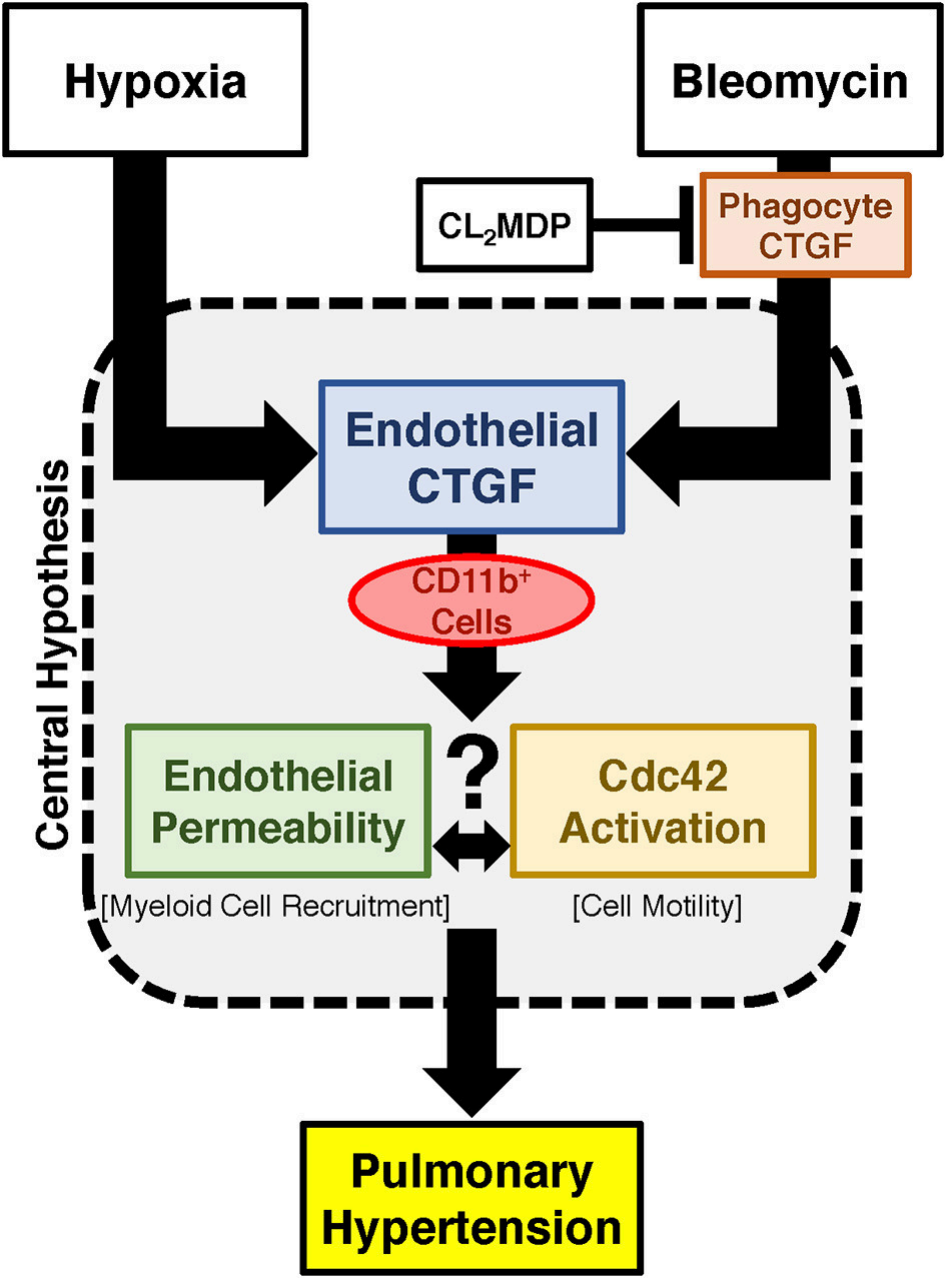
Proposed schema for development of PH regulated by endothelial cell CTGF. Endothelial CTGF contributes to recruitment of CD11b-expressing leukocytes to the lung. Cd11b^+^ cells feasibly traffic to the lung through alteration in vascular endothelial barrier function, in combination with changes in cell motility factors, such as Cdc42. Together, these factors contribute to the development of PH.

Vascular permeability is a consistent change observed in PH development (Burton et al., 2011a,b). Previous studies have shown that inhibition of vascular endothelial cell hypoxia signaling resulted in improvement in pulmonary vascular leak, associated with a decrease in CTGF production (Bryant et al., 2016). As a next step, to further investigate a potential mechanism of CTGF contribution to a known potentiator of vascular permeability and PH, we chose to examine the Rho GTPase proteins in a model of PH. This group is known to contribute to microvascular permeability (Spindler et al., 2010)—with candidate family member Cdc42 being mediated by CTGF—integrating cell polarization and migration in response to injury (Crean et al., 2004; Black and Trackman, 2008). As demonstrated in Figures 6A,B, Cdc42 activity is increased in response to both bleomycin and hypoxia treatment, and is nearly absent in whole lung samples from our eCTGF KO mice. Given difficulties with feasibility in the experimental approach of administering a daily Cdc42 inhibitor (ML141; referred to as “Cdc42i,” in figure) to mice undergoing either hypoxia exposure (frequently interrupting the development of the phenotype), or multiple injections (with bleomycin and clodronate liposome administration), and—finally—given the robust upregulation in the bleomycin model alone, we chose to test whether Cdc42 inhibition would be protective against development of bleomycin-induced PH. The administration of a Cdc42 inhibitor prevented development of PH in response to bleomycin treatment, though not to completely normal pressure measurements (Figure 6C). Importantly, pulmonary pressures were lowered in association with decreased CD11b expression (Figure 6D). Taken together, these results suggest that endothelial CTGF plays a pivotal role in inducing PH through coordinating CD11b^+^-cell trafficking to the lung due to change in induction of Cdc42 activity, which is known to influence cell polarization and motility (Summary, Figure 7).

## Discussion

Balanced regulation of wound-healing response through CTGF is a necessity for survival of an organism. Too little CTGF can impair efficiency of tissue regeneration after injury (Gibson et al., 2014), but too much can result in fibrosis and eventual organ dysfunction (Wang et al., 2011). While numerous cell-types have been found to secrete CTGF in response to chronic inflammation or injury, the tissue-specific contribution to development of aberrant vascular remodeling continues to be actively scrutinized. To this end, proper and coordinated release of CTGF by specific cells within the vascular niche, and the subsequent downstream orchestration of cell motility and proliferation, is necessary for normal vascular repair. Among the factors known to facilitate cell migration is Cdc42, which dynamically mediates actin cytoskeleton rearrangement and is directly regulated by a CTGF-integrated mechanism (Crean et al., 2004). However, the contribution of vascular endothelial CTGF to Cdc42-mediated actin polymerization and cell-cell adhesion is complex, depending on the coordinated reaction of multiple cell types within the lung. In this study, we have shown that endothelial cell expression of CTGF is necessary for development of PH secondary to chronic hypoxia, implicating a potential mechanism in the regulation of Rho family GTPase, Cdc42.

While other members of the Rho family of GTPases—such as RhoA and Rac1—have been confirmed as important in endothelial cell permeability upon exposure to noxious stimuli, the role of Cdc42 in this process remains disputed (Wojciak-Stothard et al., 2001; Fediuk et al., 2014). However, changes in endothelial cytoskeleton, adherens junctions, and permeability do correlate with Cdc42 activity in a model of hypoxia-induced neonatal pulmonary hypertension (Wojciak-Stothard et al., 2006). Importantly, CTGF works in concert with other protein mediators to increase the angiogenic response to injury, including downstream Cdc42 activation (Pi et al., 2012; Kiwanuka et al., 2016). Thus, it is interesting to speculate—based on our findings—that the spatiotemporal integration of signals that regulate cytoskeletal dynamics through CTGF, specifically Cdc42, are necessary for PH development. Future studies examining vascular endothelial models of Cdc42 up- or down-regulation upon exposure to hypoxia will help to elucidate this mechanism.

Moreover, as described above, CTGF may be directly responsible for binding to CD11b in promoting the adhesion and diapedesis of myeloid cells within inflamed vasculature (Schober et al., 2002). This finding is consistent with another report detailing that, in a model of renal fibrosis, while CTGF is expressed by myeloid cells to a slight degree, the predominant expression is in other surrounding kidney cells (Tateishi et al., 2015). Furthermore, CTGF expression is sufficient to induce bone marrow mesenchymal stem cells/stromal cells to differentiate into collagen-producing fibroblasts, contributing to fibrogenesis and pathological fibrosis (Lee et al., 2010). Interestingly, our group has recently described a contributory role of a similarly immature CD11b^+^ myeloid cell population in vascular remodeling necessary for development of chronic hypoxia-induced PH (Bryant et al., 2017). A next step in parsing out the mechanistic pathway leading to disease, will involve exploring the CTGF/CD11b interaction in *in vitro* and *in vivo* models, to understand the true molecular effect on pathophysiology of pulmonary vascular remodeling.

Though the central hypothesis is strongly supported by our experimental design—including use of our novel genetic knock out model—there are several limitations in this study worth noting. First, these data do not address a central question: what is the role of pulmonary macrophage CTGF-expression in development of pulmonary vasculopathy? As mentioned above, many different cells can express CTGF, and they often express it more strongly than macrophages. Therefore, it remains to be determined what is the relative contribution of CTGF from each of the cells toward development of PH. Though one hypothesis is that protection conferred in the bleomycin and clodronate liposome model is due to depletion of phagocyte produced CTGF, the role of those cells in the model, and the source of CTGF that drives their accumulation, requires further exploration. The contribution of other cells is also reinforced by recent studies detailing the necessary function of CTGF in bone marrow development and myeloid precursor-cell release by the stroma (Battula et al., 2013; Wang et al., 2015). Similarly, though the VECadherin Cre-recombinase is expressed primarily within vascular endothelial cells, there is slight, but potentially contributory, expression within bone marrow cells. Thus, a second caveat to the reported study is that CTGF expression may be decreased within the bone marrow stromal microenvironment in our model, resulting in inhibition of myeloid cells discharge from the developmental niche (Istvánffy et al., 2015). Such a finding would result in decreased numbers of CD11b-expressing cells in the circulation of these animals. However, contrary to this concern, we detected no gross decrease in global hematopoiesis in our murine models. Nevertheless, such a concern does emphasize the need to search for bone marrow contribution to the PH phenotype, through either generation of bone marrow chimera mice or by knocking out CTGF expression in bone marrow stromal cell populations (Cheung et al., 2014).

Overall, regulation of CTGF expression in response to injury is poorly understood on a broad spectrum encompassing potential deficits of wound repair and chronic scarring leading to fibrosis. The current study demonstrates that tissue-specific expression of CTGF by the vascular endothelium significantly influences the delicate balance between extremes of aberrant vascular wound repair, leading to development of PH. As well, we observed that inhibition of CTGF-regulated Cdc42 results in near normalization of pulmonary artery pressures. Given that vasodilator therapy use—clinically applied based primarily upon the treatment of familial/genetic pulmonary arterial hypertension—has to date failed in application to patients with Group III PH, there is a significant clinical need for evaluation of novel drug targets that meaningfully impact disease pathogenesis. We believe that endothelial cell CTGF-dependent interaction with Cdc42 in the recruitment of circulating CD11b^+^ myeloid cells is a singular example of a relevant protective pathway, possibly contributing to observed benefit in human clinical trials of anti-CTGF drugs related to pulmonary disease. Ultimately, cell-specific targeting may improve the clinical benefit of such therapies (Liu H. et al., 2006; Liu L. et al., 2006; Jung et al., 2017), with an ancillary decrease in off-target effects.

## Author contributions

LP: designed and performed experiments, analyzed data, and wrote the manuscript; CF, YL, JZ, MJ, and VS: performed experiments; KL and ES: designed experiments and edited the manuscript; AB: designed experiments, analyzed data, and edited the manuscript.

### Conflict of interest statement

KL was employed by company FibroGen, Inc. The other authors declare that the research was conducted in the absence of any commercial or financial relationships that could be construed as a potential conflict of interest.

## Supplementary material

Supplementary Figure 1 Mice without vascular endothelial cell expression of CTGF are not protected against development of pulmonary fibrosis. **(A)** Fibrosis score and **(B)** collagen content of lungs from Control and eCTGF KO mice exposed to bleomycin (*n* = 8 mice/group).

Supplementary Table 1 Antibodies used for immunostaining and flow cytometry.
